# Acute Toxicities of Proton Craniospinal Irradiation in Pediatric Medulloblastoma: A Pediatric Proton/Photon Consortium Registry (PPCR) Study

**DOI:** 10.1016/j.ijpt.2025.100747

**Published:** 2025-04-17

**Authors:** Peter Nguyen, Daniel J. Indelicato, Adrian Esterman, Arnold C. Paulino, Ralph P. Ermoian, Nadia N. Laack, Stephanie M. Perkins, Victor Mangona, Stephen Mihalcik, Jae Lee, Christine Hill-Kayser, Young Kwok, John Han-Chih Chang, John P. Perentesis, Iain MacEwan, Hien Le, Torunn I. Yock

**Affiliations:** 1 Royal Adelaide Hospital, Adelaide, Australia; 2 University of Florida Health Proton Therapy Institute, Jacksonville, Florida, USA; 3 University of South Australia, Adelaide, Australia; 4 MD Anderson Cancer Center, Houston, Texas, USA; 5 University of Washington, Seattle, Washington, USA; 6 Mayo Clinic Rochester, Rochester, Minnesota, USA; 7 Washington University in St. Louis, St. Louis, Missouri, USA; 8 Texas Center for Proton Therapy, Irving, Texas, USA; 9 Northwestern Medicine Chicago Proton Center, Warrenville, Illinois, USA; 10 ProCure New Jersey, Somerset, New Jersesy, USA; 11 University of Pennsylvania, Philadelphia, Pennsylvania, USA; 12 Maryland Proton Treatment Center, Baltimore, Maryland, USA; 13 Oklahoma Proton Center, Oklahoma City, Oklahoma, USA; 14 Cincinnati Children’s Hospital Medical Center, Cincinnati, Ohio, USA; 15 California Protons, San Diego, California, USA; 16 Massachusetts General Hospital, Boston, Massachusetts, USA

**Keywords:** Proton therapy, Medulloblastoma, Toxicity, Pediatric, Craniospinal irradiation

## Abstract

**Purpose:**

Medulloblastoma is one of the most common malignant brain tumors in children, and 75% of patients achieve long-term survival. There are limited studies reporting acute toxicity for pediatric patients receiving proton therapy for craniospinal irradiation (CSI) in medulloblastoma, and these are limited by their retrospective nature, modest cohort sizes, and lack of a comparator group.

**Materials and Methods:**

We analyzed data from the Pediatric Proton/Photon Consortium Registry. Patients with a diagnosis of medulloblastoma, receiving CSI with doses of either 23.4 Gy or 36 to 39.6 Gy, and with toxicity data at baseline and completion of treatment, were identified for inclusion.

**Results:**

A total of 272 patients were included for analysis. All patients received proton therapy. The median age of patients was 8 years (range 3-22 years), and 67.6% were male. Most patients were of good performance status with eastern cooperative oncology group (ECOG) 0 or 1, 36.8% and 31.6%, respectively. In total, 68.8% of patients had classic medulloblastoma; 76.8% had M0 disease; and 62.9% of patients had standard-risk disease.

Acute toxicities were reported as grade 1 or higher. The most common toxicities occurring during treatment were skin (90.3%), gastrointestinal (71.6%), hematological (54.9%), and mouth (32.0%). Toxicity rates were lower than in the published literature. All patients completed treatment.

**Conclusion:**

Our findings suggest that proton CSI has an acceptable degree of acute toxicity in the treatment of pediatric medulloblastoma. Future studies would benefit from toxicity grading, toxicity timing, and a photon comparator group.

## Graphical abstract

**Figure:**
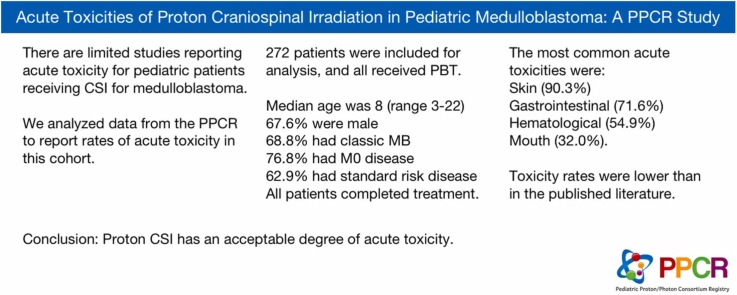

## Introduction

Medulloblastoma is one of the most common malignant brain tumors in children, accounting for 12% to 25% of all primary central nervous system tumors in the pediatric population.^1^ The peak incidence occurs in children aged 4 to 9 years.^1^ Approximately 75% of patients achieve long-term survival, but complications of treatment can have a significant adverse impact on patient-reported quality of life.^1^

Initial treatment consists of maximal safe resection of the tumor, followed by radiation therapy (RT) and chemotherapy due to the risk of metastasis through the craniospinal axis. RT is delivered to the entire craniospinal axis, and a boost is delivered to the posterior fossa or primary tumor surgical bed. Surgery, RT, and chemotherapy are all associated with significant and potentially overlapping acute and late toxicities.^1^ Given the high percentage of patients achieving long-term survival, as well as the young age of patients, methods to minimize the morbidity and toxicity of treatment are important considerations when planning treatment. A reduction in acute toxicity may also translate to reduced late toxicity in long-term survivors.

Using proton beam therapy for craniospinal irradiation (CSI) is advantageous in reducing the dose to the organs anterior to the spinal canal (thoracic and abdominopelvic viscera) and the vertebral bodies in some patients.^2^ Compared to photon (x-ray) RT, PBT can potentially reduce the volume of normal tissue irradiated by a factor of 6 to 11 when receiving CSI.^3^, ^4^ Dosimetric studies have been performed, which demonstrate a benefit to proton-based CSI compared with photon treatment, but there are few studies examining clinical outcomes.^5^, ^6^ Current studies investigating the acute toxicities experienced by patients receiving proton-based CSI are limited by their retrospective nature, modest cohort sizes, and a lack of a contemporaneous comparator group receiving photon treatment.^7^, ^8^, ^9^, ^10^, ^11^, ^12^, ^13^

## Materials and methods

### Data collection

We conducted an analysis using prospectively collected registry data from the Pediatric Proton/Photon Consortium Registry (PPCR)—an international multi-institutional consented registry of pediatric oncology patients receiving RT. Our aim was to accurately report acute toxicity data for the largest existing cohort, compare toxicity from proton-based treatment to photon-based treatment, and to determine whether patient-, clinical-, or treatment factors increased the incidence of acute toxicity.

The PPCR is an international registry established in May 2010 and is currently comprised of 24 institutions in the United States and Australia. It was established to facilitate research in outcomes related to pediatric RT treatment and is the most comprehensive multi-institutional radiation-based pediatric patient registry in existence.^14^ The registry consists of purely observational data, and participation has no impact on the treatment received.^14^ The PPCR enrolled its first patient in October 2012, and any patient receiving RT at a participating institution who is under the age of 22 is eligible. While originally limited only to patients receiving proton treatment, the protocol was amended in 2018 to include all RT modalities, including photon-based RT, particle (ion) therapy, and FLASH.^15^

The PPCR was queried for data on all patients receiving CSI from opening until mid-2021. As part of the registry, patients are reviewed at key time points in their treatment: at baseline, at the completion of treatment, and at follow-up at prespecified intervals.

Patients were selected for inclusion if they had a diagnosis of medulloblastoma, had received CSI with PBT or photon RT, received CSI doses of either 23.4 Gy or 36 to 39.6 Gy (doses used in treating standard and high-risk medulloblastoma respectively) and had documented toxicity data at baseline, and at the completion of treatment.

Study data were collected and managed using Research Electronic Data Capture (REDCap) electronic data capture tools hosted at Massachusetts General Hospital. REDCap is a secure, web-based application designed to support data capture for research studies, providing (1) an intuitive interface for validated data entry, (2) audit trails for tracking data manipulation and export procedures, (3) automated export procedures for seamless data downloads to common statistical packages, and (4) procedures for importing data from external sources.^16^

Data collected at the pretreatment baseline assessment consist of consent and registration, demographics, primary diagnosis, baseline health inventory, imaging, tumor-related surgery, and radiation information. PedsQL Questionnaires were optional. During and at the end of treatment, imaging, tumor-related surgery, radiation and chemotherapy protocol information is recorded, as well as incidence of radiation toxicity.

Toxicity recorded for patients was divided into the categories of eyes, ear, nose, mouth, cardiovascular, respiratory, gastrointestinal, genitourinary, neurological, endocrine, hematological, musculoskeletal, and skin. Toxicities within a category were reported as either present or absent, and where present, the specific toxicity was recorded from a prespecified list within REDCap. Toxicity data were not graded but designated as grade 1 or higher as per the Common Terminology for Criteria for Adverse Events (CTCAE) for comparison to the published literature.

The PPCR is a consented registry, with study approval provided by the host institutional review board at the Dana-Farber Cancer Institute at Harvard Medical School (Boston, MA, USA).^14^ Each participating institution obtains local approval from its institutional review board. Informed written consent and assent are obtained from the patient and/or their legal guardian, and each institution follows local institutional policies concerning consent, as well as reconsenting patients once they reach the age of majority.^14^ Where eligible patients choose not to consent, limited data are collected to determine whether certain demographic or diagnostic characteristics may bias against participation.^14^

### Statistical methods

Descriptive statistics were computed for clinical and treatment characteristics and the incidence of acute toxicity in each category. Toxicity could be from the tumor, the surgery, the radiation, or the chemotherapy. Where data were missing, it was excluded from the analysis. As toxicities were either present or absent, we assumed that patients who did not report toxicity at pretreatment and subsequently reported toxicity at the time of treatment assessment developed this toxicity during treatment. The incidence of these toxicities was reported separately.

Fisher exact test was used to compare categorical clinical and treatment characteristics and their relation to the incidence of acute toxicity. Values of *P* < .05 were taken as statistically significant, based on a 2-sided hypothesis.

Statistics were performed using the SPSS software package (IBM Corp Released 2020. IBM SPSS Statistics for Windows, Version 27.0. Armonk, NY: IBM Corp).

## Results

PPCR data were queried from the opening of the registry until January 2024, and 272 patients were eligible for inclusion. Patients were treated at 15 of the member institutions. Patient clinical and treatment characteristics are summarized in Table 1, Table 2, respectively.

**Table 1:** Patient clinical characteristics.

Clinical characteristic *n* = 272	*n* (%)
Age	Range: 3-22 y old
	Median: 8 y old
Gender	
Male	184 (67.6%)
Female	88 (32.4%)
Histology	
Classic	187 (68.8%)
Diffuse anaplasia/large-cell variant	42 (15.4%)
Focal anaplasia/large-cell component	17 (6.3%)
Desmoplastic or nodular variant	26 (9.6%)
Stage	
M0	209 (76.8%)
M1	5 (1.8%)
M2	21 (7.7%)
M3	37 (13.6%)
Risk category	
Standard	171 (62.9%)
Intermediate (M0, anaplastic)	30 (11.0%)
High	71 (26.1%)
ECOG	
0	100 (36.8%)
1	86 (31.6%)
2	31 (11.4%)
3	18 (6.6%)
4	2 (0.7%)
Unknown	35 (12.9%)

**Abbreviation:** ECOG, eastern cooperative oncology group.

**Table 2:** Treatment characteristics.

Treatment characteristic *n* = 272	*n* (%)
Total duration of radiation treatment	Range: 30-58 d
	Median: 42 d
Treatment delays (days) for the entire cohort	
Total delays	275
Machine disabled	56
Health-related	43
Holiday	140
Other	36
Treatment delays (number of patients)	
Total delays	142 (52.2%)
Machine disabled	45 (16.5%)
Health-related	29 (10.7%)
Holiday	97 (35.7%)
Other	25 (9.2%)
Anesthesia required	
No	112 (41.2%)
Yes	160 (58.8%)
Craniospinal irradiation dose	
23.4 Gy	187 (68.8%)
36-39.6 Gy	85 (31.3%)
Chemotherapy given	
No	12 (4.4%)
Yes	260 (95.6%)
Prior to radiation therapy	35 (12.9%)
During radiation therapy	175 (64.3%)
After radiation therapy	171 (62.9%)

The median age of patients at recruitment was 8 years (range 3-22 years). About two thirds (67.6%) of patients were male. Most patients were of good performance status, with Eastern Cooperative Oncology Group (ECOG) 0 or 1, 36.8% and 31.6%, respectively. In total, 68.8% of patients had classic medulloblastoma, and 76.8% had no evidence of metastatic disease (M0). Over half of the patients had standard-risk disease (62.9%). Thirty patients with M0 disease and large-cell/anaplastic histology were categorized as having intermediate-risk disease, and 18 of these patients received 23.4 Gy CSI, while 12 received 36 to 39.6 Gy CSI.

With respect to RT, the median overall treatment time was 42 days (range 30-58 days), with 52.2% of patients experiencing treatment delays once RT had started. Twenty-nine (10.7%) patients experienced delays due to health-related reasons, with a median overall treatment time of 44 days (range 42-58 days). Forty-five (16.5%) patients had machine-related treatment delays (median overall treatment time 43 days, range 37-58 days), while 97 (35.7%) patients had treatment breaks due to holidays (median overall treatment time 43 days, range 39-58 days). In contrast, 130 (47.8%) patients did not have any treatment delays, with a median overall treatment time of 41 days (range 30-47 days).

A total of 160 patients (58.8%) required general anesthesia for some or all treatments. The most common dose received was 23.4 Gy (68.8%), and the majority had chemotherapy as part of their treatment (95.6%), mostly concurrent (64.3%), or sequential (62.9%) following RT.

Table 3 summarizes the acute toxicities reported in each category for patients at baseline and after treatment and the percentage difference.

**Table 3:** Summary of acute toxicities reported at baseline and after treatment.

Toxicity category	Baseline	Treatment	Difference
Eye	*n* = 269	*n* = 252	
Grade 1+	123 (45.7%)	58 (23.0%)	−22.7%
Ear	*n* = 260	*n* = 242	
Grade 1+	20 (7.7%)	16 (6.6%)	−1.1%
Nose	*n* = 260	*n* = 243	
Grade 1+	9 (3.5%)	13 (5.3%)	+1.9%
Mouth	*n* = 262	*n* = 250	
Grade 1+	51 (19.5%)	80 (32.0%)	+12.5%
Cardiovascular	*n* = 260	*n* = 248	
Grade 1+	7 (2.7%)	6 (2.4%)	−0.3%
Respiratory	*n* = 259	*n* = 248	
Grade 1+	11 (4.2%)	26 (10.5%)	+6.2%
Gastrointestinal	*n* = 261	*n* = 261	
Grade 1+	85 (32.6%)	187 (71.6%)	+39.1%
Genitourinary	*n* = 256	*n* = 247	
Grade 1+	10 (3.9%)	12 (4.9%)	+1.0%
Neurological	*n* = 270	*n* = 257	
Grade 1+	216 (80.0%)	137 (53.3%)	−26.7%
Psychiatric	*n* = 260	*n* = 251	
Grade 1+	68 (26.2%)	67 (26.7%)	+0.5%
Endocrine	*n* = 259	*n* = 247	
Grade 1+	2 (0.8%)	2 (0.8%)	0.0%
Hematological	*n* = 226	*n* = 215	
Grade 1+	78 (34.5%)	118 (54.9%)	+20.4%
Musculoskeletal	*n* = 261	*n* = 248	
Grade 1+	56 (21.5%)	40 (16.1%)	−5.3%
Skin	*n* = 262	*n* = 269	
Grade 1+	46 (17.6%)	243 (90.3%)	+72.8%

At baseline, the most reported toxicity categories were neurological (80.0%), followed by eye (45.7%), hematological (34.5%), and gastrointestinal (32.6%). After treatment, the most reported toxicity categories were skin (90.3%), gastrointestinal (71.6%), hematological (54.9%), and neurological (53.3%).

Table 4 shows in detail the acute toxicities occurring during treatment. The most reported skin toxicities were alopecia (80.3%), radiation dermatitis (47.6%), erythema (35.3%), and hyperpigmentation (30.9%). The most reported gastrointestinal toxicities were nausea (52.1%), anorexia (35.6%), and constipation (30.3%). The most reported neurological toxicity was headache (23.3%). The most reported hematological toxicity was lymphopenia (44.7%), followed by low white cell count (43.3%), and anemia (39.5%). Thirty-one patients experienced thrombocytopenia (14.4%).

**Table 4:** Detailed acute toxicities reported at baseline and after treatment.

Toxicity category	Baseline	Treatment
Eye Total Blind Dry eye Eye movement disorder Eye pain/irritation Field cut Lid problems Light sensitivity Visual acuity impairment Other	*n* = 269 123 (45.7%) 1 (0.4%) 0 (0.0%) 98 (36.4%) 1 (0.4%) 1 (0.4%) 8 (3.0%) 4 (1.5%) 24 (8.9%) 15 (5.6%)	*n* = 252 58 (23.0%) 0 (0.0%) 5 (2.0%) 36 (14.3%) 6 (2.4%) 0 (0.0%) 5 (2.0%) 3 (1.2%) 11 (4.4%) 11 (4.4%)
Ear Total Ear infection Hearing impaired Tinnitus Other	*n* = 260 20 (7.7%) 0 (0.0%) 17 (6.5%) 1 (0.4%) 2 (0.8%)	*n* = 242 16 (6.6%) 1 (0.4%) 9 (3.7%) 2 (0.8%) 5 (2.1%)
Nose Total Epistaxis Nasal infection Rhinorrhea Other	*n* = 260 9 (3.5%) 2 (0.8%) 0 (0.0%) 7 (2.7%) 2 (0.8%)	*n* = 243 13 (5.3%) 2 (0.8%) 1 (0.4%) 9 (3.7%) 1 (0.4%)
Mouth Total Dysphagia Hoarseness Oral thrush Sore throat/mouth Speech impairment Vocal cord paralysis/impairment Other	*n* = 262 51 (19.5%) 22 (8.4%) 2 (0.8%) 5 (1.9%) 1 (0.4%) 32 (12.2%) 4 (1.5%) 9 (3.4%)	*n* = 250 80 (32.0%) 19 (7.6%) 1 (0.4%) 23 (9.2%) 40 (16.0%) 10 (4.0%) 2 (0.8%) 8 (3.2%)
Cardiovascular Total Cavernous malformation Conduction disorder/arrhythmia Deep vein thrombosis Hemorrhage/bleeding Hypertension Hypotension Tachycardia Other	*n* = 260 7 (2.7%) 0 (0.0%) 0 (0.0%) 0 (0.0%) 2 (0.8%) 1 (0.4%) 1 (0.4%) 1 (0.4%) 2 (0.8%)	*n* = 248 6 (2.4%) 1 (0.4%) 1 (0.4%) 1 (0.4%) 0 (0.0%) 1 (0.4%) 1 (0.4%) 2 (0.8%) 1 (0.4%)
Respiratory Total Apnea (central) Cough Dyspnea Obstructive pattern PFT Other	*n* = 259 11 (4.2%) 1 (0.4%) 4 (1.5%) 1 (0.4%) 5 (1.9%) 2 (0.8%)	*n* = 248 26 (10.5%) 0 (0.0%) 18 (7.3%) 0 (0.0%) 4 (1.6%) 10 (4.0%)
Gastrointestinal Total Anal fissure Anorexia Constipation Diarrhea Fecal incontinence Hyperphagia Lower GI bleeding Nausea Reflux Other	*n* = 261 85 (32.6%) 1 (0.4%) 18 (6.9%) 33 (12.6%) 6 (2.3%) 1 (0.4%) 1 (0.4%) 0 (0.0%) 54 (20.7%) 3 (1.1%) 1 (0.4%)	*n* = 261 187 (71.6%) 0 (0.0%) 93 (35.6%) 79 (30.3%) 8 (3.1%) 0 (0.0%) 0 (0.0%) 1 (0.4%) 136 (52.1%) 2 (0.8%) 23 (8.8%)
Genitourinary Total Polyuria Urinary incontinence Urinary tract infection Other	*n* = 256 10 (3.9%) 2 (0.8%) 4 (1.6%) 0 (0.0%) 4 (1.6%)	*n* = 247 12 (4.9%) 0 (0.0%) 5 (2.0%) 3 (1.2%) 4 (1.6%)
Neurological Total Altered mental status Ataxia Behavioral problems Coordination problems Cranial nerve impairment Developmental delay Gait disturbance (including foot drop) Headache Impaired sensation Memory impairment Neurocognitive effects on neuropsychiatric exam Neuropathy Nystagmus Paralysis (including hemiparesis or hemiplegia) Posterior fossa syndrome Seizure or seizure disorder Speech problems Weakness (side, limb, trunk) Other	*n* = 270 216 (80.0%) 3 (1.1%) 72 (26.7%) 13 (4.8%) 77 (28.5%) 50 (18.5%) 7 (2.6%) 70 (25.9%) 54 (20.0%) 4 (1.5%) 4 (1.5%) 6 (2.2%) 0 (0.0%) 29 (10.7%) 10 (3.7%) 53 (19.6%) 7 (2.6%) 60 (22.2%) 91 (33.7%) 44 (16.3%)	*n* = 257 137 (53.3%) 2 (0.8%) 40 (15.6%) 4 (1.6%) 26 (10.1%) 21 (8.2%) 1 (0.4%) 20 (7.8%) 60 (23.3%) 0 (0.0%) 1 (0.4%) 4 (1.6%) 9 (3.5%) 9 (3.5%) 3 (1.2%) 23 (8.9%) 1 (0.4%) 25 (9.7%) 52 (20.2%) 9 (3.5%)
Psychiatric Total ADD or ADHD Anxiety or anxiety disorder Depression Emotional lability Other	*n* = 260 68 (26.2%) 16 (6.2%) 26 (10.0%) 2 (0.8%) 27 (10.4%) 13 (5.0%)	*n* = 251 67 (26.7%) 6 (2.4%) 43 (17.1%) 6 (2.4%) 7 (2.8%) 18 (7.2%)
Endocrine Total Cortisol deficiency Diabetes insipidus Other	*n* = 259 2 (0.8%) 1 (0.4%) 1 (0.4%) 0 (0.0%)	*n* = 247 2 (0.8%) 0 (0.0%) 0 (0.0%) 2 (0.8%)
Hematological Total Anemia Low white cell count Lymphopenia Neutropenia Pancytopenia Thrombocytopenia Other	*n* = 226 78 (34.5%) 59 (26.1%) 37 (16.4%) 51 (22.6%) 17 (7.5%) 6 (2.7%) 12 (5.3%) 0 (0.0%)	*n* = 215 118 (54.9%) 85 (39.5%) 93 (43.3%) 96 (44.7%) 40 (18.6%) 14 (6.5%) 31 (14.4%) 2 (0.9%)
Musculoskeletal Total Edema Facial asymmetry Muscle weakness Muscular atrophy Other	*n* = 261 56 (21.5%) 1 (0.4%) 4 (1.5%) 44 (16.9%) 1 (0.4%) 13 (5.0%)	*n* = 248 40 (16.1%) 3 (1.2%) 2 (0.8%) 31 (12.5%) 0 (0.0%) 9 (3.6%)
Skin Total Alopecia Café au lait spots Dermatitis Dry skin Eczema Erythema Hyperpigmentation Rash Other	*n* = 262 46 (17.6%) 26 (9.9%) 1 (0.4%) 0 (0.0%) 1 (0.4%) 2 (0.8%) 6 (2.3%) 4 (1.5%) 8 (3.1%) 4 (1.5%)	*n* = 269 243 (90.3%) 216 (80.3%) 0 (0.0%) 128 (47.6%) 20 (7.4%) 2 (0.7%) 95 (35.3%) 83 (30.9%) 17 (6.3%) 17 (6.3%)

**Abbreviations:** ADD, attention deficit disorder; ADHD, attention deficit hyperactivity disorder; PFT, pulmonary function test.

We analyzed the impact of clinical and treatment characteristics on the incidence of acute toxicity, stratified by the presence of diffuse anaplasia/large-cell variant. Patients with anaplasia or large-cell subtype have been associated with poorer prognosis.^17^ No patients with diffuse anaplasia/large-cell variant experienced cardiovascular or endocrinological treatment toxicities.

For patients without diffuse anaplasia/large-cell variant histology: Patients of ECOG 2 to 4 performance status were more likely to experience mouth toxicity than those who were ECOG 0 to 1, 27.0% versus 44.7%, respectively (*P* = .048). Patients of ECOG 2 to 4 performance status were also more likely to experience neurological toxicity than those ECOG 0 to 1, 47.0% versus 66.7%, respectively (*P* = .035). Patients with intermediate or high-risk disease were more likely to experience genitourinary toxicities than those with standard-risk disease, 2.5% versus 11.3%, respectively (*P* = .018). Patients with metastatic disease were also more likely to experience genitourinary toxicities than those with standard-risk disease, 3.0% versus 11.1%, respectively (*P* = .039). Patients with intermediate or high-risk disease were more likely to experience psychiatric toxicities than those with standard-risk disease, 22.8% versus 37.0% (*P* = .049). Patients with ECOG 2 to 4 were more likely to experience musculoskeletal toxicities than those with ECOG 0 to 1, 12.2% versus 26.3% (*P* = .041).

For patients with diffuse anaplasia/large-cell variant histology, patients with M0 disease were more likely to experience mouth toxicities than those with metastatic disease, 55.6% versus 16.7% (*P* = .037). Patients who experienced a health-related delay were more likely to experience respiratory toxicities than those who had no delays, 12.5% versus 60.0% (*P* = .037). Patients with ECOG 0 to 1 performance status were more likely to experience gastrointestinal toxicities, 82.1% versus 33.3% (*P* = .031).

## Discussion

This is the largest multi-institutional analysis of acute toxicity data for pediatric patients with medulloblastoma receiving CSI, which has been shown to result in acceptable rates of acute toxicity. Song et al^12^ and Brown et al^18^ were the first to publish studies comparing acute toxicities experienced by patients receiving proton and photon CSI for medulloblastoma in the pediatric and adult populations, respectively. There are limited studies reporting acute toxicities of CSI, consisting primarily of retrospective cohort studies or case series. Liu et al^19^ published the largest study of 97 patients (60 receiving proton therapy) but only reported on hematological toxicity. Yock et al^9^ reported a nonrandomized phase 2 trial investigating long-term toxic effects of proton RT and reported acute toxicity for 59 patients undergoing proton RT for pediatric medulloblastoma, and this provided the basis for comparison for nonhematological toxicities.

We found that the number of patients reporting eye, ear, cardiovascular, neurological, and musculoskeletal toxicities was lower after treatment than at baseline, and this could be attributed to recovery after primary debulking surgery. In contrast, more patients reported nose, mouth, respiratory, gastrointestinal, genitourinary, hematological, and skin toxicities after treatment.

A significant limitation in our data is the lack of uniformity in treatment follow-up data collection, which occurred at different time points, including after completion of CSI, after posterior fossa/primary tumor surgical bed boost, and possibly after chemotherapy administration.

We reported grade 1 or higher alopecia and radiation dermatitis in 80.2% and 47.6% of patients, respectively, which is lower than in Yock et al’s^9^ study, where all patients experienced these toxicities. This is lower than expected, given that the advantage of PBT is in the reduction of exit dose-entrance dose through posteriorly-directed RT fields is typically higher than photon treatment due to the absence of the skin-sparing effect. We expected that all patients would experience grade 1 or higher alopecia and radiation dermatitis during treatment, and differences are likely to be due to under-reporting of this toxicity since it is a highly expected outcome of the whole brain component of treatment.

After treatment, 71.6% of patients reported acute gastrointestinal toxicity, with 32.6% reporting this toxicity at baseline. 52.1% of patients developed nausea during treatment, which is similar to the 54% to 60% reported by Yock et al.^9^ Interpretation of this result is again limited by the timing of assessment, as nausea and vomiting can be a result of CSI, posterior fossa irradiation, or chemotherapy.

The incidence of grade 1 or higher hematological toxicity of proton CSI ranges widely in the published literature, with the majority being grade 3 or less.^9^, ^19^ A major concern regarding acute hematological toxicity from CSI is the potential to delay treatment due to the need for supportive measures, or to prevent patients from completing their prescribed course of treatment.^20^, ^21^ Half the patients in our cohort experienced treatment delays due to machine breakdown, public holidays, or patient health, and all completed RT treatment. The patients with health-related treatment delays had a longer overall treatment time compared to the rest of the cohort, and this has been associated with inferior overall survival.^22^

Vertebral-body-sparing RT has been proposed as a method of reducing hematological toxicity but was previously limited to adult patients due to concerns that dose gradients across the vertebral bodies of pediatric patients would lead to deformity.^10^ A small case series performed by MacEwan et al^10^ found that the use of vertebral-body-sparing CSI did not appear to cause increased severe spinal abnormalities, suggesting that this could be another approach to reducing acute toxicity. Hashimoto et al^13^ found a lower incidence of serious acute hematological toxicity for patients receiving proton CSI, and 4 patients in their study received vertebral-body-sparing treatment. They suggest that further studies are required to evaluate the differences in hematological toxicity due to differences in co-administered chemotherapy agents and scheduling, and differences in doses delivered to the vertebral body.^13^ Data were not available for patients in our cohort, but in another analysis of the PPCR, 18.6% of patients who were skeletally immature (boys aged <15, and girls aged <13) received vertebral-body-sparing CSI.^23^

The higher incidence of oral thrush and sore throat is likely to be attributable to the use of chemotherapy, as 95.6% of our patients received chemotherapy as part of their treatment. The dose received by the pharynx and oral cavity is typically lower compared to patients receiving photon-based craniospinal or posterior fossa boost.

The strengths of this study include the large sample size, the international multi-institutional cohort, and the prospectively collected data for each patient. One of the limitations of this study is the lack of a photon comparator group, as all patients received proton treatment. While the PPCR has been expanded to include all radiation treatment modalities, most member institutions primarily treat patients with PBT. We expect that as more institutions join the PPCR, the number treated with photons and other treatment modalities will increase. While a randomized controlled trial would provide an ideal comparator cohort, this study design has been deemed to be unfeasible and unethical.^24^, ^25^

Our study is also limited by the lack of toxicity grading, uncertainty regarding the timing of toxicity, and incomplete or erroneous registry data. Toxicity data were recorded as a binary data point, and where a toxicity was present, there was scope for “free text” to further describe the toxicity. The lack of grading using a tool such as the CTCAE limited comparison to previous published studies, which all used the contemporaneous version of CTCAE.^7^, ^8^, ^9^, ^10^, ^12^, ^13^, ^19^

Toxicity data for the treatment time point are documented as being recorded at completion of treatment.^14^ This introduces difficulty when determining the contribution of each treatment modality to the toxicity experienced. Headache, nausea, and hematological toxicity could be accounted for by CSI, but also by the posterior fossa boost phase of RT, as well as chemotherapy given during or after RT. There was also limited information available regarding the chemotherapy treatment regimen received by patients, although the majority of medulloblastoma patients receive vincristine as the standard of care, although during this era some high-risk patients also received daily carboplatin—but that was usually in the context of the Children’s Oncology Group study ACNS0332 which is closed and published demonstrating that only the children with high-risk disease and group 3 medulloblastomas benefited from daily concurrent carboplatin (Leary, JAMA Onc, 2021). Previous studies have addressed this by excluding patients receiving concurrent chemotherapy or by only including patients receiving single-agent vincristine, an agent with limited hematological toxicity.^12^, ^19^

## Conclusion

In conclusion, this is the largest study reporting on the acute toxicities experienced by pediatric patients receiving proton CSI for the treatment of medulloblastoma. The most common toxicities associated with proton CSI were alopecia, radiation dermatitis, nausea and vomiting, and hematological toxicity. Our results were consistent with or compared favorably to published toxicity data for this setting, but generalizability is limited by the absence of toxicity grading. Our findings suggest that PBT has a very acceptable degree of acute toxicity, with 10.9% of patients experiencing a health-related treatment delay, not all of which are necessarily attributable to the RT. Importantly, all patients completed their treatment.

Future studies reporting acute toxicity data would benefit from grading toxicity according to a standardized tool for both clinician-scored and patient-reported outcome measures, as well as the stringent recording of the timing of acute toxicity to better understand the contribution of each treatment modality. We expect that studies using PPCR data in the future will benefit from an increase in the number of member institutions and enrollment of patients having nonproton treatment as a contemporaneous comparator group.

## Funding

The PPCR is supported by the Federal Share of program income earned by Massachusetts General Hospital on C06 CA059267, Proton Therapy Research and Treatment Center. No funding was received for the writing of this publication.

## Author Contributions

Conceptualization: P.N., H.L. and T.I.Y.; Data curation: P.N.; Formal analysis: A.E.; Investigation: D.J.I., A.C.P., R.P.E., N.N.L., S.M.P., V.M., S.M., J.L., C.H., Y.K., H.H.C., J.P.P., I.M. and T.I.Y.; Methodology: H.L. and A.E.; Supervision: H.L. and T.I.Y.; Writing - original draft: P.N., H.L., and T.I.Y.; Writing - review & editing: P.N., H.L., D.J.I., A.C.P., R.P.E., N.N.L., S.M.P., V.M., S.M., J.L., C.H., Y.K., J.H.C., J.P.P., and I.M.

## Declaration of Conflicts of Interest

The authors declare the following financial interests/personal relationships which may be considered as potential competing interests: Torunn I Yock reports a relationship with MIM Software Inc that includes funding grants. John Perentesis reports a relationship with Varian Medical Systems Inc that includes funding grants. Daniel Indelicato reports a relationship with the National Cancer Institute that includes speaking and lecture fees. Daniel Indelicato reports a relationship with Ronald McDonald House Charities that includes board membership. Stephanie Perkins reports a relationship with Mevion Medical Systems that includes speaking and lecture fees. Stephanie Perkins reports a relationship with Mevion Medical Systems that includes board membership. Victor S. Mangona reports a relationship with Texas Oncology that includes equity or stocks. Hien Le reports a relationship with the Flinders Foundation that includes funding grants. Hien Le reports a relationship with the Royal Australian and New Zealand College of Radiologists that includes funding grants. Hien Le reports a relationship with Channel Seven Children’s Research Foundation Inc that includes funding grants. Hien Le reports a relationship with the Pancreatic Cancer Research Fund that includes funding grants. Hien Le reports a relationship with the Australian Cancer Research Foundation that includes funding grants. Hien Le reports a relationship with Neurosurgical Research Foundation that includes funding grants. Hien Le reports a relationship with the Cooperative Research Centers Program that includes funding grants. Hien Le reports a relationship with AstraZeneca that includes speaking and lecture fees. Hien Le reports a relationship with the Royal Australian and New Zealand College of Radiologists that includes board membership. Hien Le reports a relationship with Royal Adelaide Hospital that includes board membership and employment. Hien Le reports a relationship with TROG Cancer Research that includes board membership. If there are other authors, they declare that they have no known competing financial interests or personal relationships that could have appeared to influence the work reported in this paper.

## References

[1] Young S., Phaterpekar K., Tsang D.S., Boldt G., Bauman G.S. (2023). Proton radiotherapy for management of medulloblastoma: a systematic review of clinical outcomes. Adv Radiat Oncol.

[2] Ruggi A., Melchionda F., Sardi I. (2022). Toxicity and clinical results after proton therapy for pediatric medulloblastoma: a multi-centric retrospective study. Cancers.

[3] Yock T.I., Tarbell N.J. (2004). Technology insight: proton beam radiotherapy for treatment in pediatric brain tumors. Nat Clin Pract Oncol.

[4] Hoffman K.E., Yock T.I. (2009). Radiation therapy for pediatric central nervous system tumors. J Child Neurol.

[5] Yoon M., Shin D.H., Kim J. (2011). Craniospinal irradiation techniques: a dosimetric comparison of proton beams with standard and advanced photon radiotherapy. Int J Radiat Oncol Biol Phys.

[6] Miralbell R., Lomax A., Cella L., Schneider U. (2002). Potential reduction of the incidence of radiation-induced second cancers by using proton beams in the treatment of pediatric tumors. Int J Radiat Oncol Biol Phys.

[7] Suneja G., Poorvu P.D., Hill-Kayser C., Lustig R.A. (2013). Acute toxicity of proton beam radiation for pediatric central nervous system malignancies. Pediatr Blood Cancer.

[8] Yuh G.E., Loredo L.N., Yonemoto L.T. (2004). Reducing toxicity from craniospinal irradiation: using proton beams to treat medulloblastoma in young children. Cancer J Sudbury Mass.

[9] Yock T.I., Yeap B.Y., Ebb D.H. (2016). Long-term toxic effects of proton radiotherapy for paediatric medulloblastoma: a phase 2 single-arm study. Lancet Oncol.

[10] MacEwan I., Chou B., Moretz J., Loredo L., Bush D., Slater J.D. (2017). Effects of vertebral-body-sparing proton craniospinal irradiation on the spine of young pediatric patients with medulloblastoma. Adv Radiat Oncol.

[11] Liu I.C., Holtzman A.L., Rotondo R.L. (2021). Proton therapy for adult medulloblastoma: acute toxicity and disease control outcomes. J Neurooncol.

[12] Song S., Park H.J., Yoon J.H. (2014). Proton beam therapy reduces the incidence of acute haematological and gastrointestinal toxicities associated with craniospinal irradiation in pediatric brain tumors. Acta Oncol Stockh Swed.

[13] Hashimoto T., Shimizu S., Takao S. (2019). Clinical experience of craniospinal intensity-modulated spot-scanning proton therapy using large fields for central nervous system medulloblastomas and germ cell tumors in children, adolescents, and young adults. J Radiat Res.

[14] Kasper H.B., Raeke L., Indelicato D.J. (2014). The pediatric proton consortium registry: a multi-institutional collaboration in U.S. proton centers. Int J Part Ther.

[15] Lawell M.P., Indelicato D.J., Paulino A.C. (2020). An open invitation to join the Pediatric Proton/Photon Consortium Registry to standardize data collection in pediatric radiation oncology. Br J Radiol.

[16] Harris P.A., Taylor R., Thielke R., Payne J., Gonzalez N., Conde J.G. (2009). Research electronic data capture (REDCap)--a metadata-driven methodology and workflow process for providing translational research informatics support. J Biomed Inform.

[17] Rutkowski S., von Hoff K., Emser A. (2010). Survival and prognostic factors of early childhood medulloblastoma: an international meta-analysis. J Clin Oncol.

[18] Brown A.P., Barney C.L., Grosshans D.R. (2013). Proton beam craniospinal irradiation reduces acute toxicity for adults with medulloblastoma. Int J Radiat Oncol Biol Phys.

[19] Liu K.X., Ioakeim-Ioannidou M., Susko M.S. (2021). A multi-institutional comparative analysis of proton and photon therapy-induced hematologic toxicity in patients with medulloblastoma. Int J Radiat Oncol Biol Phys.

[20] Kumar N., Miriyala R., Thakur P. (2017). Impact of acute hematological toxicity on treatment interruptions during cranio-spinal irradiation in medulloblastoma: a tertiary care institute experience. J Neurooncol.

[21] Jefferies S., Rajan B., Ashley S., Traish D., Brada M. (1998). Haematological toxicity of cranio-spinal irradiation. Radiother Oncol J Eur Soc Ther Radiol Oncol.

[22] Baliga S., Bajaj B.V.M., Kabarriti R. (2020). Prolongation of radiotherapy duration is associated with inferior overall survival in patients with pediatric medulloblastoma and central nervous system primitive neuroectodermal tumors. Pediatr Blood Cancer.

[23] Connor M., Paulino A.C., Ermoian R.P. (2022). Variation in proton craniospinal irradiation practice patterns in the United States: a Pediatric Proton Consortium Registry (PPCR) study. Int J Radiat Oncol.

[24] Wolden S.L. (2013). Protons for craniospinal radiation: are clinical data important?. Int J Radiat Oncol Biol Phys.

[25] Johnstone P.A.S., McMullen K.P., Buchsbaum J.C., Douglas J.G., Helft P. (2013). Pediatric CSI: are protons the only ethical approach?. Int J Radiat Oncol Biol Phys.

